# A Retrospective Analysis of Patients' Conditions Using Acupuncture in a Traditional Korean Medicine Hospital

**DOI:** 10.1155/2015/103683

**Published:** 2015-10-01

**Authors:** Kyung-Jin Yun, Ju Ah Lee, Jiae Choi, Mi Mi Ko, Cham-kyul Lee, Myeong Soo Lee, Eun-Yong Lee

**Affiliations:** ^1^Department of Acupuncture & Moxibustion Medicine, Chung-Ju Hospital of Traditional Korean Medicine, Semyung University, Chung-Ju 27427, Republic of Korea; ^2^KM Fundamental Research Division, Korea Institute of Oriental Medicine, Daejeon 305-811, Republic of Korea; ^3^Clinical Research Division, Korea Institute of Oriental Medicine, Daejeon 305-811, Republic of Korea

## Abstract

*Objective. *The aim of this study was to identify the patient demographics, health issues, and type of acupuncture treatments who visited a traditional Korean medical hospital for acupuncture treatment. *Methods. *We retrospectively analysed the data using the electronic medical records (EMRs) of patients treated with at least one treatment of acupuncture from 1 January 2010 to December 2012 in the Chung-Ju Korean hospital at Semyung University. *Results.* The total number of identified patients was 1189 inpatients and 10138 outpatients. The 50–59 age group received acupuncture treatment in the hospital the most, followed by the 40–49 age group. Among the patients undergoing acupuncture treatment because of a diagnosis of pain, 82.74% were outpatients and 72.85% were inpatients. Additionally, all patients with a spine condition received acupuncture treatment. The most common musculoskeletal conditions of patients at the traditional Korean medicine (TKM) hospital were associated with spine conditions, such as low back pain and neck pain. Various treatments have been performed at the hospital in conjunction with acupuncture. The study results show a high prevalence of acupuncture treatment for diagnosed diseases. *Conclusion.* Our study suggests the need to investigate additional TKM hospitals to analyse characteristics of patients who received specific treatments. Analysis of the characteristics of patients treated with Korean acupuncture at the TKM hospital in this study will help future researchers who want to implement strong clinical evidence. However, we cannot completely discount all symptoms because of the retrospective nature of this study, and only one hospital was used, which limits the generalisation of our findings.

## 1. Introduction

Recently, the use of complementary and alternative medicine (CAM) has grown in popularity worldwide. A study showed that 38.3% of American adults and 52.2% of Australians use some form of CAM [1]. And various researches about CAM use, attitude, and awareness have been conducted. One study reported 6-year comparative economic evaluation of healthcare costs and mortality rates of Dutch patients from conventional and CAM GPs [2]. And one study revealed awareness, use, attitude, and perceived need for complementary and alternative medicine (CAM) education among undergraduate pharmacy students in Sierra Leone [3]. And another study studied CAM Use and Suggestions for Medical Care of Senior Citizens [4].

However, very little is known about the current state of clinical practice in detailed individual CAM treatment. CAM has many diverse treatment methods: acupuncture, moxibustion, cupping and herbal therapy, and so forth. Many traditional Korean medicine (TKM) hospitals use electronic medical records (EMRs), so information is available to analyse characteristics of patients who have received CAM treatment.

Acupuncture is a CAM technique that is used for treating a variety of conditions, especially pain conditions [5]. According to National Health Insurance data from Korea, pain was the major reason for an annual acupuncture consultation [6]. In the US and UK, the most frequent CAM users were also patients who suffered from back pain or osteoarthritis [7]. A study showed that CAM utilisation is most strongly associated with musculoskeletal disorders [8].

Another study analysed EMR charts and showed that CAM usage was strongly associated with musculoskeletal disorders and pain conditions [9].

Therefore, the aim of this study was to provide information on the characteristics of patients who received acupuncture treatment in an academic hospital using a large sample size and long study duration (3 years).

## 2. Methods

### 2.1. Study Design

The medical records of patients treated with acupuncture who received at least one session of acupuncture at the Chung-Ju TKM hospital at Semyung University between 1 January 2010 and 31 December 2012 were retrospectively reviewed.

### 2.2. Data Sources

The information abstracted from EMRs included the age, gender, total number of outpatients and inpatients, total number of outpatient visits and admission days for inpatients, the name and frequency of diagnosis, frequencies of received acupuncture, and other ancillary interventions using a predefined excel spread sheet (Microsoft; Redmond, Washington, USA).

Diagnosis codes of symptoms were classified into 14 categories of major issues based on the site of symptoms to conduct a more clinically relevant analysis and better interpret the data. We used International Statistical Classification of Diseases and Related Health Problems 10th revision (ICD-10) as selecting method of patient records. Also, we measured the total costs of outpatient and inpatient management per patient.

### 2.3. Study Criteria

Patients who were treated as inpatients and outpatients at the TKM hospital were included.

The same patient could be duplicated because he or she could have been treated for new pains or discomforts. Therefore, if the same patient was treated for a different diagnosis code, we considered this case to be a new patient.

The exclusion criteria included the following: patients with full private insurance coverage, such as traffic accident patients, patients treated in an emergency room, patients who paid a discounted price because of a signed Memorandum of Understanding (MOU), and patients treated only by herbal medicine to promote health or physical therapy. We only used EMR data; therefore, the visit schedules of patients were not required.

### 2.4. Data Synthesis

A descriptive statistical analysis was conducted. Continuous data are presented as the means and SDs, and categorical data are presented as percentage and frequency. Continuous data with an asymmetrical distribution are presented as the medians and a percentile range (25th and 75th percentiles). In calculating the mean and SD of the number of sessions per patients, we divided the total sessions by the number of patients. The data were statistically analysed with SAS software, version 9.1.3 (SAS Institute Inc., Cary, NC).

*Cost Analysis*. We extracted data from the EMRs which are about patient-paid expenses for outpatient and inpatient care government-paid insurance reimbursements and per patient.

### 2.5. Ethical Considerations

An institutional review board at the Semyung University Chung-Ju TKM hospital approved this study (number 1310-06). IRB waived the receiving of informed consent of individual patients because the electronic records in this study were used anonymously.

## 3. Results

### 3.1. The Patient Demographics, Health Issues, and Type of Acupuncture Treatments Provided

The descriptive statistics for patient characteristics are listed in Table 1. The total number of patients was 11327. Of the patients identified, most patients visited the TKM hospital as outpatients (10138/11327, 89.5%). The total number of patients was 11327, including 10138 outpatients and 1189 inpatients. The mean patient age was 48.64 years among outpatients and 49.43 years among inpatients. The percentage of men was 53.82 in outpatient and 42.47 in inpatient. The percentage of women was 46.17 in outpatient and 57.52 in inpatient.

Regardless of inpatient or outpatient status, most patients were aged 40–59 years (outpatient 53.51% and inpatient 46.60%).

### 3.2. TKM Treatments

Diverse acupuncture styles were used, and patients were treated with multiple interventions. All patients (outpatients and inpatients) received manual stimulation of the needle (*n* = 11327; 100%). And electroacupuncture was used in 69.66% of outpatients and 34.15% of inpatients, and pharmacoacupuncture was used in 15.63% of outpatients and 40.79% of inpatients. Direct moxibustion was used in 0.49% of outpatients and 0.67% of inpatients, whereas indirect moxibustion was used in 2.17% of outpatients and 27.67% of inpatients.

In total, 10138 outpatients received 43767 acupuncture treatments. Acupuncture treatments were performed with an average of 4.32 times per patient. Manual acupuncture plus Infrared (98.57%) was the most used method, followed by electroacupuncture (69.66%), wet cupping (43.13%) and herbal medicine (21.02%).

Each inpatient received an average of 28.23 acupuncture treatments, and a total of 1189 inpatients received 33570 acupuncture treatments. Manual acupuncture plus cupping (77.71%) and herbal medicine (75.95%) were the most performed treatments, followed by Infrared (50.97%), pharmacoacupuncture (40.79%), and electroacupuncture (34.15%) (Table 2).

### 3.3. Medical Conditions

The diagnostic analysis of acupuncture treatments is shown in Table 3. Approximately 82.74% of outpatients and 72.85% of inpatients receiving acupuncture treatment were diagnosed with pain. Additionally, all patients with spine condition received acupuncture treatment. The most treated conditions of outpatients were lower back pain (26.69%), neck pain (14.28%), and shoulder pain (11.67%).

The most treated conditions of inpatients were also lower back pain, miscellaneous (21.84%), and neck pain (14.55%). 5471 outpatients and 2556 inpatients had two different diagnosis codes (Table 3).

*Medical Costs*. The median total cost for outpatient and inpatient care per patient was 80,122 and 1,112,360 Korean Won (Table 4).

## 4. Discussion

The present study analysed patients who received acupuncture therapy at a TKM hospital by using EMRs. This result is also consistent with previous studies that showed low back pain and lumbar sprain as the most treated conditions in TKM hospitals or Korean clinics [9]. The Scottish Intercollegiate Guideline Network (SIGN) recommended acupuncture for the management of chronic pain with an “A” grade of recommendation. This recommendation means that acupuncture should be considered for short term relief of pain in patients with chronic low back pain [10].

This study revealed that both men and women aged 50–59 years visited the TKM hospital most frequently and men visited the TKM hospital slightly more than women. However, previous research has demonstrated that female patients use TKM hospitals more than men [9]. This difference may be because of regional differences between two hospitals. Additionally, pervious research has focused on a descriptive analysis of patients undergoing pain management, and this study focused on patients who wanted to receive acupuncture therapy.

Acupuncture is usually used in conjunction with various TKM interventions, including infrared, electroacupuncture, wet cupping, herbal medicine, and pharmacoacupuncture, in Korean clinics.

A high use of herbal medicine was observed in inpatient care, which suggests that herbal medicine may be regarded as an essential element of treatment in inpatient management.

The number of acupuncture treatments was high among inpatients compared with outpatients because of the severity of conditions. Among outpatients, the reason for the low proportion of direct and indirect moxibustion was the time limits of patients. The low usage of moxibustion in outpatients may be because of time limits as well.

The limitations of this study include the following. First, we collected all diagnoses of each patient. Therefore, chief complaints or the primary diagnosis of patients was not considered. Most patients had one or two diagnoses, but several had many diagnoses.

Additional information related to patient characteristics, such as pain duration or symptom severity or satisfaction of treatment and cure rate, could not be collected because of limited resources. Therefore, we only showed the characteristics of people who received acupuncture treatment; the results could not provide useful information.

Therefore, only descriptive data were available. Additionally, we could not extract the exact acupuncture points and techniques of ancillary treatments because these data were not extractable from the EMRs. However, we identified that acupuncture was treated with other interventions mostly, and also we identified the real cost of how much patients paid for CAM treatment. This paper will be helpful in the designing of clinical trials or cost effectiveness research. Although several limits exist, this study could be utilised in TKM research as a foundation to identify the characteristics of TKM.

In conclusion, our study suggests the need to investigate additional TKM hospitals to analyse characteristics of patients who received specific treatments. Analysis of the characteristics of patients treated with Korean acupuncture at the TKM hospital in this study will help future researchers who want to implement strong clinical evidence. However, we cannot completely discount all symptoms because of the retrospective nature of this study, and only one hospital was used, which limits the generalisation of our findings.

In the future, it has to be conducted in several hospitals and more researches of cost effectiveness or prospective observational study should be conducted.

## Figures and Tables

**Table 1:** Demographic characteristics of patients treated with TKM medical hospital from 2010 to 2012 (*n* = 11327).

Classification	Outpatients (*N* = 10138)		Inpatients (*N* = 1189)	
	*n*	%	*n*	%
Number of patients	10138	89.5	1189	10.49
Sex				
Male	5457	53.82	505	42.47
Female	4681	46.17	684	57.52
Mean age (years)^*∗*^	48.64	14.71	49.43	16.99
Age group (years)	Male/female (*n*)	Male/female (%)	Male/female (*n*)	Male/female (%)
>30	568/471	5.6/4.6	75/79	6.3/6.6
30–39	970/569	9.6/5.6	115/69	9.7/5.8
40–49	1464/1162	14.4/11.5	124/153	10.4/12.9
50–59	1462/1337	14.4/13.2	110/167	9.3/14.0
60–69	610/6344	6.0/6.3	33/73	2.8/6.1
70–79	312/408	3.1/4.0	38/108	3.2/9.1
80<	71/100	0.7/1.0	10/35	0.8/2.9
Total	10138	100	1189	100

Values are provided as the ^*∗*^mean (SD) or *n* (%) where appropriate.

**Table 2:** Total number and distribution of acupuncture treatment received for 3 years.

	Outpatients (*N* = 10138)			Inpatients (*N* = 1189)		
	Total sessions	Number of patients (%)	Mean sessions per patient^**∗**^	Total sessions	Number of patients (%)	Mean sessions per patient^**∗**^
Acupuncture						
Manual acupuncture	43767	10138 (100)	4.32 (7.87)	33570	1189 (100)	28.23 (35.24)
Electroacupuncture	30104	7062 (69.66)	4.26 (7.51)	3787	406 (34.15)	9.33 (14.56)
Pharmacoacupuncture	8793	1585 (15.63)	5.55 (9.71)	3308	485 (40.79)	6.82 (11.60)
Manual acupuncture plus cupping						
Wet cupping	16454	4373 (43.13)	3.76 (5.92)	9244	924 (77.71)	10.01 (9.73)
Dry cupping	50	11 (0.11)	4.55 (5.41)	198	20 (1.68)	9.90 (8.50)
Manual acupuncture plus moxibustion						
Direct mox.	315	50 (0.49)	6.30 (9.59)	58	8 (0.67)	7.25 (7.19)
Indirect mox.	822	220 (2.17)	3.74 (5.47)	4535	329 (27.67)	13.77 (17.08)
Manual acupuncture plus chuna	504	146 (1.44)	3.45 (3.95)	173	71 (5.97)	2.44 (2.04)
Manual acupuncture plus herb	65567	2131 (21.02)	30.77 (28.84)	37592	903 (75.95)	41.63 (51.17)
Manual acupuncture plus IR	45691	9993 (98.57)	4.27 (7.86)	3763	606 (50.97)	6.21 (10.72)

IR: Infrared, Values are presented as *n* (%) or mean (SD).

^*∗*^Mean session = number of patients/total session.

**Table 3:** Classification of medical symptoms of patients in acupuncture treatment.

Diagnosis	Outpatients (*N* = 10138)	Inpatients (*N* = 1189)
	*n* (%)	*n* (%)
Pain		
Spine pain (lumbar)	4166 (26.69)	1188 (31.72)
Spine pain (neck)	2229 (14.28)	545 (14.55)
Spine pain (back, thoracic, and rib)	426 (2.73)	133 (3.55)
Spine pain (etc.)	382 (2.45)	156 (4.17)
Shoulder pain	1822 (11.67)	185 (4.94)
Upper extremity pain (excluding shoulder)	1383 (8.86)	107 (2.86)
Pelvic pain	261 (1.67)	53 (1.41)
Hip/knee pain	909 (5.82)	156 (4.17)
Lower extremity pain	301 (1.93)	76 (2.03)
Ankle/foot pain	881 (5.65)	79 (2.11)
Multiple site pain	153 (0.98)	50 (1.34)
Other conditions		
Facial disease	653 (4.18)	135 (3.60)
Headache	358 (2.29)	64 (1.71)
Etc.	1685 (10.80)	818 (21.84)
Total	**15,609**	**3745**

Values are provided as *n* (%)

Patients received multiple treatments according to each symptom classification.

**Table 4:** Costs for the outpatient and inpatient care per patient for 3 years.

Medical costs (per patient)	Outpatients	Inpatients
Government-paid cost	29,620 KRW (16840, 62200)	602,690 KRW (282548, 1142094)
Patient-paid cost	41,806 KRW (22202, 132408)	455,074 KRW (130000, 1014330)
Total expenditure	**80,122 KRW (40992, 209428)**	**1,112,360 KRW (570287, 2080160)**

Data are presented as the medians and percentile ranges (the 25th and 75th percentiles). Exchange rate assumes that 1 £ is worth 1732 KRW.

KRW, Korean Won.
